# Book Reviews

**Published:** 1895-10

**Authors:** 


### *BOOK REVIEWS.*

Practical Dietetics. With Special Reference to Diet in Disease. By W. Gilman Thompson, M.D., Professor of Materia Medica and Clinical Medicine in the University of the City of New York, etc. Illustrated. Price $5.00. Published by D. Appleton & Co., 72 Fifth Avenue, New York.

This book will fill one of the few remaining "long-felt wants." The subject of the dietetic treatment of disease has not received the attention which it deserves. It forms no part of the courses in college curricula, and text-books on the practice of medicine usually dismiss it with some such expression as "The patient should be carefully fed," or "A proper and nutritious diet is of the first importance." This work is intended to remedy these shortcomings and to furnish the practitioner a text-book containing instructions as to the appropriate diet in diseases which are influenced by right feeding.

Beginning with the elementary composition of foods, the author next discusses the force-producing value of different foods and the comparative nutritive properties of vegetable and animal foods, etc. In the section on animal foods much attention is given to milk in all the forms in which it is used for food. This is one of the best portions of the work.

The author considers the general relations of food to special diseases; those that are caused by dietetic errors and the administration of food for the sick, giving the necessary rules as to method, time, etc. Dietetic treatment in fever in general is followed by instructions for diet in specified diseases, with lists of food suitable for the patient, in certain stages of the disease, as in the infectious fevers and other acute affections.

The feeding of pregnant women, nursing mothers, infants, and young children constitutes a very important part of the work, and an appendix contains receipts for invalid food and beverages suitable for fevers and convalescence from acute illness.

This book gives evidence of wide knowledge and intelligent observation on the part of the author, and we think it is an important and valuable addition to medical literature. The practitioner will find it highly useful, containing much that can be found nowhere else, so far as we know.

---

SKIASCOPY, AND ITS PRACTICAL APPLICATION TO THE STUDY OF REFRACTION. By Edward Jackson, A.M., M.D., Professor of Diseases of the Eye in the Philadelphia Polyclinic, etc. Published by The Edwards & Docker Co., Philadelphia. Price $1.

The need of such a work on a subject so necessary for every-day refractive work is evident to every ophthalmologist, and no one is so well qualified for elucidating this subject of skiascopy as Dr. Jackson. The purport of the work is better understood by quoting the preface of its author: "This little book was written to bring about the more general adoption of skiascopy as an essential part of the examination for ametropia. It is not supposed that any ophthalmologist is quite ignorant of the test; but many do not know its full practical value or how best to apply it."

The little book is complete in every detail, while the mathematical and analytical mind, with which Dr. Jackson is blessed, has elucidated in a practical manner those points which are usually the most difficult to comprehend. The importance of *skiascopy*, or as it is sometimes called *retinoscopy*, in arriving at correct errors of refraction by the ophthalmologist, cannot be overestimated. No conscientious ophthalmologist should prescribe glasses for a person under the age of forty without having subjected his patient to this test. It is a most admirable little book. D. R.

---

DISORDERS OF THE MALE SEXUAL ORGANS. By Eugene Fuller, M.D., Instructor in Genito-Urinary and Venereal Diseases in New York Post-Graduate Medical School, etc. Published by Lea Brothers & Co., 706–710 Sansom street, Philadelphia.

The title of this book is very misleading, embracing more than is described in the text. It is a monograph on acute and chronic seminal vesiculitis, and should have been so named. It is an elaboration of several papers which the author has published on the subject within late years (*Journal Cutaneous and Venereal Diseases*, September, 1893, June and July, 1894). It is a description of the anatomy, physiology, and pathology of the seminal vesicles, with the clinical features, diagnosis, and treatment of inflammatory conditions of these parts. The author thinks that disease of the seminal vesicle is not recognized as often as it should be, and in his opinion many of those cases of persistent urethral discharges which cause so much trouble to both patient and doctor are due to involvement of the vesicles. "Stripping the vesicles" with the finger in the rectum is the proper direct treatment, with constitutional remedies in particular cases. The last chapter describes several illustrative cases. The book is well worthy a careful reading.

---

THE SCIENCE AND ART OF OBSTETRICS. By Theophilus Parvin, A.M., M.D., LL.D., Professor of Obstetrics and Diseases of Women and Children, Jefferson Medical College, etc. Third edition, carefully revised. Illustrated with 269 Woodcuts and two Colored Plates. Lea Brothers & Co., Philadelphia.

We have just received the third edition of this book, which thoroughly maintains the high standard of the other two editions. It has been largely rewritten and additional illustrations have been introduced. The book is divided into two parts; the first part is subdivided into three sections, which gives a section each to the physiology of pregnancy, labor, and the puerperal state. Part second is also divided into three sections, which give the pathology of each of these conditions. The section on physiology of pregnancy gives the anatomy and physiology of the organs and also the management of pregnancy, etc. The other sections of the book are equally thorough. We take pleasure in recommending the book to practitioners and students. Dr. Parvin's work on obstetrics, we think, is easily the equal of any in the English language.

J. L. C.

PATHOLOGY AND MORBID ANATOMY. By T. Henry Green, M.D., Lecturer on Pathology and Morbid Anatomy at Charing-Cross Hospital Medical School, London. Seventh American from the eighth and revised English edition. Octavo volume of 595 pp., with 224 engravings, and a colored plate. Cloth, $2.75. Philadelphia, Lea Brothers & Co., Publishers, 1895.

This is described as an introduction to the above subject.

The *Introduction* to the matter to follow deals with the *Normal Cell*, its characters, physiology, development. Then *Disease*, its varieties, etiology, effects, terminations.

Then follow chapters upon *Nutrition Arrested*, *Nutrition Impaired*, and *Nutrition Increased*, with the conditions appertaining thereto classified according to the head under which they belong.

*Tumors* of the various kinds are next treated of, and then the *Diseases* of the *Blood* and *Circulation; Inflammation.* Next come *Vegetable Parasites*, then the *Infective Granulomata; Septicæmia* and *Pyæmia; Malaria; Diseases* of *Special Tissues* and *Organs* occupy several chapters, and the book ends with *Pathology* of the *Central Nervous System*. The cuts are most excellent. M. B. H.

---

PHYSIOLOGICAL FACTORS OF THE NEUROSES OF CHILDHOOD. By B. K. Rachford, M.D., Professor of Physiology and Clinician to Children's Clinic, Medical College of Ohio, etc. Price $1.00. Published by The Robert Clarke Company, Cincinnati, Ohio.

This little book is a republication of the series of papers which have appeared under the same title in the *Archives of Pediatrics*. These papers have been highly worthy of close study, giving evidence of much careful research. It is a thoughtful discussion of those local and general nervous disorders which do not depend on known local pathological lesions of the nervous system. The author has made a very interesting and instructive little volume.

